# A Cross-Sectional Study of SARS-CoV-2 Seroprevalence between Fall 2020 and February 2021 in Allegheny County, Western Pennsylvania, USA

**DOI:** 10.3390/pathogens10060710

**Published:** 2021-06-06

**Authors:** Lingqing Xu, Joshua Doyle, Dominique J. Barbeau, Valerie Le Sage, Alan Wells, W. Paul Duprex, Michael R. Shurin, Sarah E. Wheeler, Anita K. McElroy

**Affiliations:** 1 Division of Infectious Diseases, Department of Pediatrics, School of Medicine, University of Pittsburgh, Pittsburgh, PA 15261, USA; lix16@pitt.edu (L.X.); JDOYLE@pitt.edu (J.D.); DJB176@pitt.edu (D.J.B.); 2 Center for Vaccine Research, University of Pittsburgh, Pittsburgh, PA 15261, USA; pduprex@pitt.edu; 3 Department of Microbiology and Molecular Genetics, University of Pittsburgh, Pittsburgh, PA 15261, USA; valerie.lesage@pitt.edu; 4 Department of Pathology, School of Medicine, University of Pittsburgh, Pittsburgh, PA 15261, USA; wellsa@upmc.edu (A.W.); shurinmr@upmc.edu (M.R.S.); Wheelerse3@upmc.edu (S.E.W.); 5 Department of Immunology, University of Pittsburgh, Pittsburgh, PA 15261, USA

**Keywords:** SARS-CoV-2, seroprevalence, ELISA, neutralization assay

## Abstract

Seroprevalence studies are important for understanding the dynamics of local virus transmission and evaluating community immunity. To assess the seroprevalence for SARS-CoV-2 in Allegheny County, an urban/suburban county in Western PA, 393 human blood samples collected in Fall 2020 and February 2021 were examined for spike protein receptor-binding domain (RBD) and nucleocapsid protein (N) antibodies. All RBD-positive samples were evaluated for virus-specific neutralization activity. Our results showed a seroprevalence of 5.5% by RBD ELISA, 4.5% by N ELISA, and 2.5% for both in Fall 2020, which increased to 24.7% by RBD ELISA, 14.9% by N ELISA, and 12.9% for both in February 2021. Neutralization titer was significantly correlated with RBD titer but not with N titer. Using these two assays, we were able to distinguish infected from vaccinated individuals. In the February cohort, higher median income and white race were associated with serological findings consistent with vaccination. This study demonstrates a 4.5-fold increase in SARS-CoV-2 seroprevalence from Fall 2020 to February 2021 in Allegheny County, PA, due to increased incidence of both natural disease and vaccination. Future seroprevalence studies will need to include the effect of vaccination on assay results and incorporate non-vaccine antigens in serological assessments.

## 1. Introduction

Severe Acute Respiratory Syndrome Coronavirus 2 (SARS-CoV-2) spread quickly and caused a worldwide pandemic, affecting social and economic life globally [1]. Diagnosis of SARS-CoV-2 acute infection relies on viral tests such as PCR or virus antigen detection. However, these tests lack the ability to identify prior infections. In contrast, serological assays such as enzyme-linked immunosorbent assays (ELISAs) measure antibody responses to specific virus antigens and are useful for determining the prevalence of a disease in an affected area and can identify individuals as potential donors for convalescent plasma therapeutics [2]. 

All current Emergency Use Authorization (EUA)-authorized serological tests for SARS-CoV-2 target the nucleocapsid (N) or spike (S) protein. N protein facilitates the replication of viral RNA and the assembly and release of viral particles after infection [3]. S protein binds to the angiotensin-converting enzyme 2 (ACE2) receptor on the surface of human cells for cell entry of SARS-CoV-2 [4,5]. The receptor-binding domain (RBD) of S protein is a main target of anti-viral antibodies [6]. Both S and N proteins are highly expressed during infection and are immunogenic [6,7]. Two leading mRNA-based SARS-CoV-2 vaccines, one developed by Moderna and the other by Pfizer and BioNTech, both use S protein as an immunogen [8,9].

Population-based seroprevalence studies of COVID-19 carried out in hotspots of COVID-19 across the world between March and June 2020 showed a 4.41% seroprevalence in the US and a 3.38% seroprevalence worldwide [10,11,12]. One nationwide study conducted between July and September 2020 showed a range of 1–23% jurisdiction-level seroprevalence and an estimate of fewer than 10% people with detectable SARS-CoV-2 antibodies, indicating that the majority of the US population had not yet been exposed [13]. Although evidence-based information about the efficacy of COVID-19 interventions is urgently needed in all communities, most studies so far have focused on large scale populations either nationwide or in metropolitan areas and less is known about seroprevalence in medium-sized cities. Freeman et al. reported a seroprevalence of 1% in the first half of 2020 in the immunocompromised pediatric patients in one pediatric quaternary care center in Pittsburgh, PA [14]. However, data for the general population in this area are lacking. 

The goal of this study was to develop a serological testing strategy to estimate the seroprevalence of SARS-CoV-2 in the population of Allegheny County, Western PA, in Fall 2020 and February 2021, which are two critical time points before and after the large wave of cases that occurred between December 2020 and January 2021, as well as the introduction of two EUA-approved vaccines in US in Dec 2020. In addition, we used two ELISA assays that enabled us to distinguish infected from vaccinated individuals (DIVA), which allowed for a further comparison of demographics between infected and vaccinated individuals. Overall, we observed a 4.5-fold increase in SARS-CoV-2 seroprevalence from Fall 2020 to February 2021 in Allegheny County, PA, by RBD ELISA. Among samples positive for RBD, 36.4% from Fall 2020 and 66.7% from February 2021 were also positive by neutralization assay. These changes were driven by both natural disease acquisition and vaccination rollout. Additionally, our data showed income and race disparities in infection and vaccination, respectively, suggesting the need to better support people of disadvantaged groups during the pandemic.

## 2. Results

### 2.1. SARS-CoV-2 RBD and N ELISA Validation

RBD and N ELISA assays had a limit of detection of 1:100. Specificity of the SARS-CoV-2 ELISA assays was determined using 183 human serum samples collected before the pandemic [15]. This included healthy volunteers, as well as patients who tested positive for other viral or inflammatory conditions, including other human coronaviruses (See Supplementary Table S1. Legend for details). 

**Figure 1:**
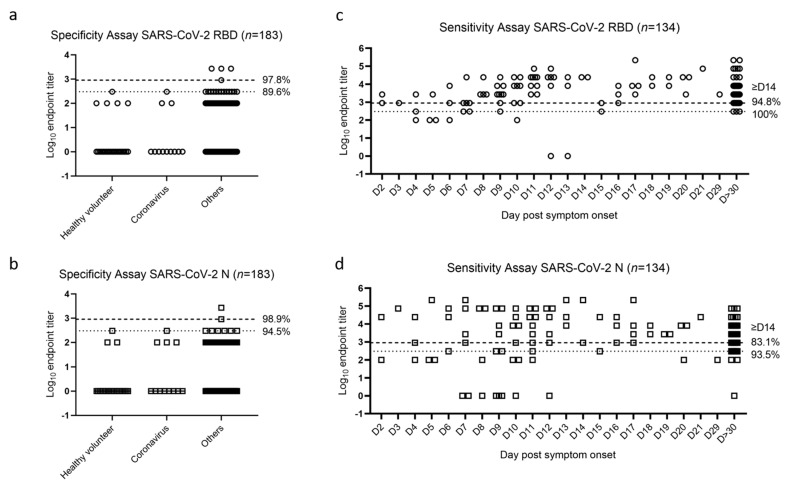
Specificity and sensitivity analysis of RBD and N ELISAs. Each circle represents one sample for RBD (**a**,**c**) and each square represents one sample for N (**b**,**d**) in each assay. Samples were grouped by positivity for a specific infection or assay (**a**,**b**) and see Supplemental Table S1 for details of “Others”. Samples from patients with COVID-19 disease are grouped by the day post-self-reported-symptom onset (**c**,**d**). Dashed line indicates a titer at 900 and dotted line indicates a titer at 300. Numbers next to each line represents the specificity or sensitivity of the assay at the given cutoff value. Samples collected on or after Day 14 post symptom onset were used for sensitivity analysis.

With an endpoint titer cutoff of 300, the assays had 89.6% (RBD) or 94.5% (N) specificity. With an endpoint titer cutoff of 900, the assays had 97.8% (RBD) or 98.9% (N) specificity (Figure 1a,b). Sensitivity of the assays was determined using 134 human serum samples from patients known to have COVID-19 between 16 March 2020 and 12 May 2020 [15]. In some cases, multiple samples from an individual patient were available on different days post symptom onset. Samples collected on or after Day 14 post symptom onset were used for sensitivity analysis. In this cohort, there was 100% (RBD) and 93.5% (N) sensitivity at an endpoint titer cutoff of 300, and 94.8% (RBD) and 83.1% (N) sensitivity at an endpoint titer cutoff of 900 (Figure 1c,d). An endpoint titer cutoff of 900 was chosen for use in the RBD assay and an endpoint titer cutoff of 300 was chosen for use in the N assay, with a specificity at 97.8% for RBD and 94.5% for N, and a sensitivity at 94.8% for RBD and 93.5% for N. RBD and N ELISAs were further validated using WHO international standards (Supplementary Figure S1).

### 2.2. Seroprevalence of COVID-19 in Allegheny County, Western PA 

A total of 199 human blood samples were collected in the Fall of 2020 and 194 samples in February of 2021. A total of 88.5% of the study subjects were from Allegheny County and 9.9% were from other counties in PA (Supplementary Table S2). Women, seniors, and African Americans were more represented in the study cohort compared to that in the population of Allegheny County (Supplementary Table S2). 

The endpoint titers for both RBD and N were determined for each sample and using predefined cutoffs, the seroprevalence of SARS-CoV-2 in the Fall cohort was 5.5% by RBD ELISA, 4.5% by N ELISA, and 2.5% for both. In the February cohort, the seroprevalence increased to 24.7% by RBD ELISA, 14.9% by N ELISA, and 12.9% for both (Table 1).

**Figure 2:**
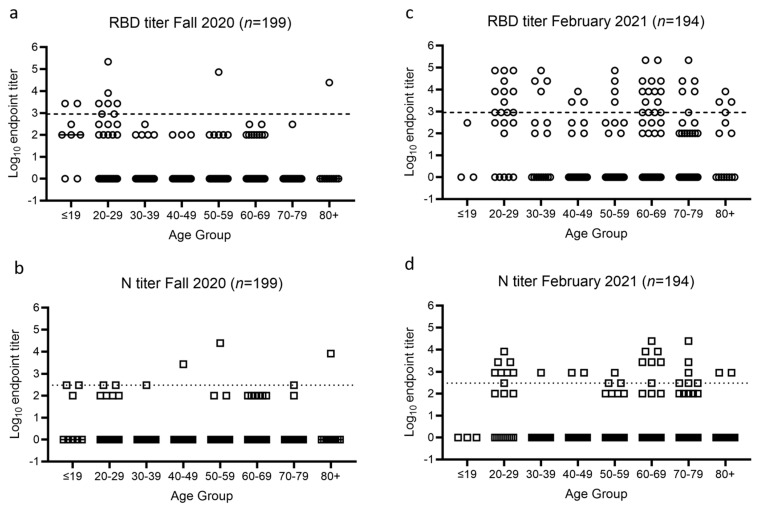
RBD and N endpoint titers of all samples. Each circle represents one sample for RBD (**a**,**c**) and each square represents one sample for N (**b**,**d**) at the two time points of the study. Dashed line indicates a titer at 900 as cutoff for RBD positive and dotted line indicates a titer at 300 as cutoff for N positive. Samples are grouped by age.

The 11 samples positive for RBD in Fall 2020 included two documented recovered cases of COVID-19 (one male aged 50–59 and one female aged 80+) and two volunteers who were enrolled in the Phase III clinical trial of the Moderna vaccine (Figure 2a). 

The remaining seven samples positive for RBD in Fall 2020 were all from young adults aged 19–29, several of which had reported a history of either contact with COVID-19 confirmed cases, or mild COVID-19 related symptoms, but were never tested. This suggested a higher local prevalence of COVID-19 in young adults in Fall 2020 (Figure 2a). 

**Figure 3:**
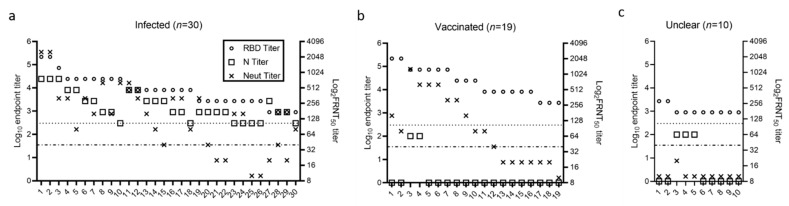
Titer comparison between RBD ELISA, N ELISA, and Neutralization assay. RBD endpoint titer, N endpoint titer, and FRNT_50_ titer of all RBD positive samples in groups of infected (**a**), vaccinated (**b**), and unclear (**c**). Left y axis is Log_10_ endpoint titers for RBD and N ELISAs. Right y axis is Log_2_FRNT_50_ titer. Dotted line indicates a titer at 300 which was the cutoff titer for N positive. Dash-dotted line indicates a titer of 40 as cutoff for positive neutralization.

In the February cohort, the increased seroprevalence suggested increases in either acquisition of natural disease during the winter peak or vaccination following EUA for BNT162b2 by Pfizer and BioNTech on 11 December 2020 and mRNA-1273 by Moderna on 18 December 2020 (Table 1). Both BNT162b2 and mRNA-1273 are S protein mRNA-based vaccines, so vaccination would be expected to elicit RBD antibodies but not N antibodies. In contrast, infection by SARS-CoV-2 would trigger immune responses against both RBD and N. Therefore, based on their RBD and N titers, all 59 individuals who tested positive for RBD were separated into three groups, infected (*n* = 30, RBD+ and N+), vaccinated (*n* = 19, RBD+ and N−), or unclear (*n* = 10 RBD+ and N−) (Figure 3). These classification groups were informed by self-reporting or chart review. The group designated as unclear had RBD titers that were either at or one dilution above the endpoint titer cutoff value and had no corroborating data from chart review or self-reporting. 

**Figure 4:**
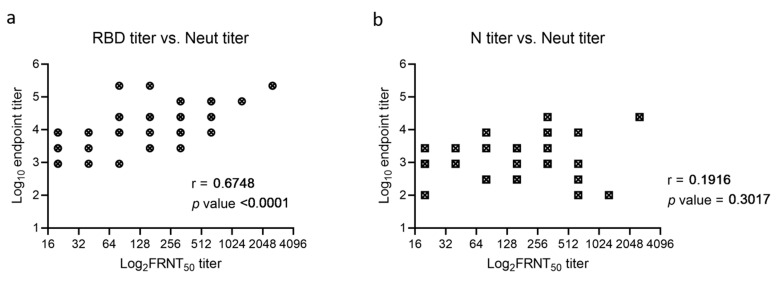
Correlation between ELISA titer and Neutralization titer. Samples from Figure 3 with titers above the detection threshold (100 for RBD and N, 20 for FRNT_50_) of each assay were selected for the correlation analysis between neutralization titer and RBD titer (**a**) or N titer (**b**). Spearman’s Rank Correlation Coefficient r and Probability (p) value (two-tailed) are shown.

All RBD-positive samples were tested in an FRNT_50_ assay. The neutralization assay had a limit of detection of 1:20, and samples were considered positive if the titer was ≥40. Among the 30 individuals classified as infected, 24 (80%) were positive by FRNT_50_; the 6 that were unable to neutralize had an RBD titer ≤2700 (Figure 3a). In comparison, among the 19 individuals classified as vaccinated, 12 (63.2%) were positive by FRNT_50_; the 7 that were unable to neutralize had an RBD titer ≤8100 (Figure 3b). All unclear samples failed to neutralize the virus, even though they had a positive RBD titer (Figure 3c). Comparison between FRNT_50_ and ELISA titers revealed a significant correlation for RBD but not N (Figure 4a,b). Notably, samples with RBD titers at 8100 and positive N titers were more often able to neutralize SARS-CoV-2, than samples with RBD titers at 8100 but negative N titers, suggesting that despite having the same RBD titer, there might be a qualitative difference in spike antibodies generated during infection versus vaccination. 

### 2.3. Comparison of Income and Race between Infected and Vaccinated Groups 

Demographic comparisons were evaluated for age, income, and race in infected (*n* = 25) and vaccinated (*n* = 17) individuals from the February 2021 cohort. In both groups, the average age was approximately 50. The median household income of participants was significantly lower in the infected group than that of the vaccinated group (Supplementary Figure S2a). Three students were removed from this comparison since their income data were felt to be reflective of their guardians’ income as their zip codes were out of state. The race distribution between infected and vaccinated groups in the February cohort revealed similar percentages of African American and White in the infected group but a lower percentage of African American in the vaccinated group (Supplementary Figure S2b). These data are consistent with other reports demonstrating the disproportionate effects of the pandemic and vaccine accessibility on lower-income and African American groups in PA as well as nationwide US [16,17,18,19]. 

## 3. Discussion

The ELISAs used in this study were shown to have a high degree of sensitivity and specificity. These assays are semi-quantitative while most commercially available antibody detection kits only offer qualitative results. The ability to perform functional, neutralizing assays of antibody activity remains limited to BSL-3 laboratory settings. Therefore, an assay such as the RBD ELISA, which is significantly correlated with neutralization titer, has utility for measuring virus-specific activity and could possibly be used to define a correlate of immune protection in clinical and vaccine studies. We observed a 4.5-fold increase in community immunity by RBD ELISA between Fall of 2020 and February of 2021 and were able to demonstrate that this increase was driven both by infection and vaccination. 

The correlations between RBD, N, and neutralization were particularly interesting. When a serum sample had a high RBD titer (≥24,300), which indicated strong immune responses elicited by either infection or vaccination, that sample had the ability to neutralize the virus regardless of the N titer. Out of 12 samples with RBD titers at 900, only 2 were positive in the neutralization assay, both of which also had a positive N titer. Two samples with negative RBD titers but positive N titers (one at 900 and the other at 2700) were evaluated for neutralization and neither of them neutralized SARS-CoV-2 virus, suggesting these were false positives or possibly they represent cross reactivity with another coronavirus [20]. When a sample had an RBD titer between 900 and 24,300, it appeared more likely to neutralize the virus if it also had a positive N titer. A total of 12 out of 16 samples from infected individuals with an RBD titer at 2700 or 8100 demonstrated neutralization, whereas only 2 out of 8 samples from vaccinated individuals did. This could have several implications. First, the fact that 4 out of 16 samples with positive RBD and N titers failed to neutralize the virus was consistent with previous findings that people who had contracted COVID-19 could sometimes be reinfected [21,22]. Secondly, the complete vaccination history was not available for all samples, therefore it is possible that some vaccinated cases were not fully vaccinated, meaning that they either received one dose or received the second dose within 14 days at the time of sample collection. Lastly, two infected samples with RBD titer = 2700 and N titer = 300 demonstrated high neutralization titer at 160, suggesting that other factors may also play a role in the generation of neutralizing activity. For example, binding sites on the ACE2 receptor and the binding affinity of an antibody have both been shown to be critical for neutralization potential [23,24,25]. The SARS-CoV-2 immune response is different in infected versus vaccinated individuals [26]. This could result in a greater breath or alter the class switching and/or affinity maturation of S-specific antibodies in infected versus vaccinated individuals and is consistent with our observations. 

The entire population was naïve to SARS-CoV-2 infection when this virus emerged. However, the pandemic has affected people from diverse social and economic backgrounds differently. By comparing income and race distribution between infected and vaccinated groups, we found that people with lower incomes were more likely to have been infected than vaccinated and people of color were less likely to have been vaccinated at the time of sampling. The February 2021 cohort of specimens were obtained when PA restricted vaccination to health care workers, residents/care staff in skilled nursing facilities, individuals ages 65 and older and those ages 16–64 with certain underlying medical conditions [27]. Restrictions on access to vaccination could have influenced these results. 

This study has several limitations. Although people of both genders and most age groups and races were included, it had more women, seniors (aged 60–69 and 70–79), and African Americans as compared to the representative distribution in Allegheny County, PA (Supplementary Table S2). In addition, whereas children under 15 make up 15.6% of the population, this study was focused on adults. Finally, patient information regarding previous COVID-19 diagnosis or vaccination was limited to data accessible by chart abstraction for the majority of participants. A major strength of this study is the use of two assays that permits classification of individuals as either vaccinated or infected. With many of the vaccine platforms containing the S antigen, the need for a non-vaccine antigen for community serosurveys of infection is of increased importance. In addition, estimating local seroprevalence using a small cohort of samples can be a valuable tool for quickly assessing the degree of immunity in a community and can inform public health decisions during a pandemic. 

## 4. Materials and Methods

### 4.1. Human Samples

Human subjects research was performed according to University of Pittsburgh approved IRB protocols (20030228 and 20040220). Positive and negative control samples used for specificity and sensitivity assays were from residual samples at UPMC clinical laboratories and have been previously described [15]. WHO international standards were from the National Institute for Biological Standards and Control (NIBSC). Serum or plasma was collected and stored at −20 °C.

Blood samples obtained in Fall 2020 were from two cohorts. One cohort included 100 samples obtained between 21 September 2020 and 13 October 2020 from healthy adults who were willing to have phlebotomy performed for infectious disease research. All participants self-reported their age, sex, race, ethnicity, residential zip code, history of travel and immunization, as well as history of known COVID-19 disease or contact with COVID-19 confirmed cases. A second cohort of 99 samples were residual specimens obtained from the UPMC Mercy clinical laboratory between 2 November 2020 to 4 November 2020; these specimens were from outpatients undergoing routine bloodwork. Blood samples from February 2021 were all residual specimens obtained from the UPMC Mercy outpatient laboratory between 1 February 2021 and 5 February 2021. Age, sex, race, residential zip code and any history of COVID-19 disease or testing was abstracted from the medical record prior to de-identification for all UPMC Mercy outpatient samples.

### 4.2. Enzyme-Linked Immunosorbent Assay (ELISA) 

MaxiSorp^TM^ 96-well plates (Thermofisher) were coated with SARS-CoV-2 RBD-His or SARS-CoV-2 N-His protein (see Supplementary Material for details on clone construction and protein purification) at 50 ng per well diluted in PBS and incubated at 4 °C overnight. Following removal of coating solution, plates were blocked with blocking buffer (5% non-fat milk in PBS with 0.1% Tween-20, PBST) and incubated at 37 °C for 1 h. Three-fold serial dilutions of samples were prepared in blocking buffer, then incubated on plates at 37 °C for 2 h. Plates were washed three times with PBST followed by incubation with donkey anti-human IgG horseradish peroxidase (HRP) conjugated secondary antibody (Jackson ImmunoResearch) diluted 1:10,000 in blocking buffer at 37 °C for 1 h. Plates were washed again prior to the addition of TMB peroxidase substrate mix (Seracare) and incubated at room temperature (RT) for 5 min. TMB stop solution (Seracare) was added and the optical density (OD) at 450 nm was measured using a Molecular Devices SpectraMax 340PC Microplate Reader. A normal human serum non-reactive to SARS-CoV-2 was included as negative control and a human serum known to be reactive to SARS-CoV-2 was included as positive control on each plate. 

### 4.3. Focus Reduction Neutralization Test (FRNT) 

Neutralization assays were performed as previously described [28] except with 2 × 10^3^ FFU/mL of SARS-CoV-2 (University of Pittsburgh clinical isolate from June 2020). Fixation was performed using 4% paraformaldehyde for 20 min at RT. Rabbit anti-SARS-CoV-2 N antibody (GenScript custom, 1:3000 diluted in blocking buffer) was used as primary antibody and goat anti-rabbit IgG HRP (Jackson ImmunoResearch, 1:1000 diluted in blocking buffer) was used as secondary antibody. MossBio TMB substrate was used for foci development. The FRNT_50_ is the dilution of sera that neutralized at least 50% of the input virus.

### 4.4. Data Analysis

The US Census 2019 ACS 5-year estimates of the population in Allegheny County, PA, was accessed online and data regarding race, age, sex and median household income based upon zip code was abstracted. All graphs were made using GraphPad Prism Version 9. Correlation coefficient (r) and two-tailed probability (p) values were calculated using Spearman’s correlation. Confidence intervals were calculated using the Clopper–Pearson exact method. The endpoint titer was defined as the dilution of the serum that gave an OD value at least three standard deviations above the average value obtained from the negative control serum. 

## Figures and Tables

**Table 1:** Analysis and comparison of seroprevalence and antibody neutralization between Fall 2020 and February 2021.

	Fall 2020 (*n* = 199)	February 2021 (*n* = 194)
RBD positive (endpoint titer ≥ 900)—No. (%) [95%CI]	11 (5.5) [3–10]	48 (24.7) [19–31]
N positive (endpoint titer ≥ 300)—No. (%) [95%CI]	9 (4.5) [2–8]	29 (14.9) [10–21]
RBD and N both positive—No. (%) [95%CI]	5 (2.5) [1–6]	25 (12.9) [9–18]
Neut positive (FRNT_50_ ≥ 40)—No. (%) [95%CI]	4 (2.0) [1–5]	32 (16.5) [12–22]

## Data Availability

Data is contained within the article or supplementary material.

## Supplementary Materials

The following are available online at https://www.mdpi.com/article/10.3390/pathogens10060710/s1, Figure S1: Comparison of RBD and N titers between the in-house assays and that reported by NIBSC using WHO international standards. Five dots shown on each graph represent samples of one healthy donor collected before 2019 and four COVID-19 recovered patients. Spearman’s rank correlation coefficient r and probability (*p*) value (two-tailed) are shown. Figure S2: Comparison of income and race between infected and vaccinated cases in February 2021. Median income distribution in infected and vaccinated groups (a): Y axis is the median household income of the area based on the zip code of the home address reported by each study subject. The line in each group represents the median. * represents a *p* value < 0.05 by an unpaired *t*-test (two-tailed). Race distribution in infected and vaccinated groups (b): Total samples refer to all samples collected in February 2021. Data were compared to that of American Community Survey (ACS) 5-year estimates for Allegheny County, PA, generated by United States Census Bureau. Table S1: Detailed groups of samples used in the Specificity Assays of SARS-CoV-2 RBD and N. Table S2: Demographics of study subjects.
