# Supplementary material for: A Cross-Sectional Study of SARS-CoV-2 Seroprevalence between Fall 2020 and February 2021 in Allegheny County, Western Pennsylvania, USA
Source: Pathogens. 2021 Jun 6;10(6):710. doi: 10.3390/pathogens10060710 (PMC8226606; doi:10.3390/pathogens10060710)
Supplement: Supplementary file 1 [file pathogens-10-00710-s001.zip › pathogens-1236271-supplementary.pdf]

Supplemental Materials and Methods:

Production and purification of SARS-CoV-2 RBD protein

The pCAGGS SARS-CoV-2 RBD-His construct (a gift from the Krammer lab) was transfected into FreeStyle™ 293-F cells using 293fectin™ Transfection Reagent (Thermo Scientific). Supernatants from transfected cells were harvested on day 3 post-transfection and SARS-CoV-2 RBD-His protein was purified using Pierce™ Ni-NTA magnetic Agarose Beads (ThermoFisher) following the manufacturer's protocol. Purity was confirmed by SDS-PAGE followed by staining with SimplyBlue SafeStain (ThermoFisher) and protein was quantitated using a Bradford assay.

Construction of pET-20b(+) SARS-CoV-2 N-His plasmid and production and purification of SARS-CoV-2 N protein

The SARS-CoV-2 N (GeneID: 43740575) was amplified from viral RNA and cloned into pET-20b(+) vector using In-Fusion® HD Cloning Kit. Primers were: forward primer for pET-20b(+): CACCACCACCACCACCACTG; reverse primer for pET-20b(+): CTCGAGTGCGGCCGCAAG; forward primer for SARS-CoV-2 N-His: GCGGCCGCACTCGAGATGTCTGATAATGGACCCCAAAATC; reverse primer for SARS-CoV-2 N-His: GTGGTGGTGGTGGTGGGCCTGAGTTGAGTCAGCAC. pET-20b(+) SARS-CoV-2 N-His was transformed into BL21(DE3)pLysS cells, cultured in medium supplemented with 100ug/ml Ampicillin and 34μg/ml Chloramphenicol (LB-Amp-Chl) at 37°C overnight with agitation. The following day, the starter culture was inoculated into 250mL LB-Amp-Chl and incubated at 37°C with agitation until the OD reached 0.6-1.0. IPTG (0.4uM) was added for 4-6h. Cells were centrifuged and lysed using BugBuster® Master Mix (Millipore Sigma) following the protocol for inclusion body purification after BugBuster® Master Mix treatment (Novagen pET system manual 11th edition), except both the supernatant and inclusion bodies were used. N was purified using Pierce™ Ni-NTA magnetic Agarose Beads (ThermoFisher) following the manufacturer's protocol. Purity was confirmed by SDS-PAGE followed by staining with SimplyBlue SafeStain (ThermoFisher) and protein was quantitated using a Bradford assay.

Supplemental Table 1. Detailed groups of samples used in the Specificity Assays of SARS-CoV-2 RBD and N

	RBD titer >=900	RBD titer >=300	N titer >=900	N titer >=300	Number of samples
Healthy volunteer	0	1	0	1	24
Coronavirus	0	1	0	1	12
Others					
EBV	0	0	0	0	6
VZG	0	0	0	0	8
HSV1	0	0	0	0	1
HSV1+HSV2	0	0	0	0	1
HSV1+EBV	0	0	0	0	2
HSV2+EBV	0	0	1	1	1
HSV1+HSV2+EBV	0	1	1	1	2
HSV1+HSV2+VZG	0	0	0	0	1
HSV1+EBV+VZG	0	0	0	0	2
HSV2+EBV+VZG	0	0	0	0	1
CMV	2	2	0	1	11
TOXG	0	0	0	0	1
TOXG+CMV	0	0	0	0	2
TOXG+VZG	0	0	0	0	1
SYPHT	0	0	0	0	4
RPR	0	0	0	0	7
RUB	0	0	0	0	1
RPR+RUB	0	0	0	0	2
SYPHT + RPR	0	1	0	0	8
HCV	0	0	0	0	10
HBV	0	0	0	0	5
HCV+HBV	0	0	0	0	1
ANA	0	4	0	2	18
HIV5G	1	2	0	2	19
RVPVE	1	7	0	1	32
Total	4	19	2	10	183
Specificity (%)	97.8%	89.6%	98.9%	94.5%	

Abbreviations: **EBV**: Epstein-Barr Virus. **VZG:** Varicella-Zoster Virus IgG. **HSV1**: Herpes Simplex Virus 1 IgG. **HSV2**: Herpes Simplex Virus 2 IgG. **CMV**: Cytomegalovirus. **TOXG**: *Toxoplasma gondii* IgG. **SYPHT**: Syphilis total antibodies. **RPR**: Rapid Plasma Reagin antibodies screening test for Syphilis. **RUB**: Rubella. **HCV**: Hepatitis C Virus IgG. **HBV**: Hepatitis B Virus IgG. **ANA**: Antinuclear Antibodies. **HIV5G**: Human Immunodeficiency Virus IgG. **RVPVE**: Respiratory viral panel positive.

Supplemental Table 2. Demographics of study subjects

	Fall 2020 (n=199)	Spring 2021 (n=194)	Total (n=393)	US Census 2019 ACS 5-Year Estimates, Allegheny County
**Geographics - no. (%)**				
Allegheny County, PA	181 (91.0)	167 (86.1)	348 (88.5)	
Other counties, PA	17 (8.5)	22 (11.3)	39 (9.9)	
Other states	1 (0.5)	5 (2.6)	6 (1.5)	
**Sex - no. (%)**				
Female	124 (62.3)	108 (55.7)	232 (59.0)	631,402 (51.7)
Male	75 (37.7)	86 (44.3)	161 (41.0)	590,342 (48.3)
**Age - no. (%)**				
under 15	0 (0.0)	1 (0.5)	1 (0.3)	190,020 (15.6)
15-19	8 (4.0)	2 (1.0)	10 (2.5)	71,850 (5.9)
20-29	48 (24.1)	21 (10.8)	69 (17.6)	172,473 (14.1)
30-39	27 (13.6)	17 (8.8)	44 (11.2)	165,882 (13.6)
40-49	16 (8.0)	20 (10.3)	36 (9.2)	139,138 (11.4)
50-59	27 (13.6)	30 (15.5)	57 (14.5)	167,937 (13.7)
60-69	38 (19.1)	55 (28.4)	93 (23.7)	160,204 (13.1)
70-79	25 (12.6)	33 (17.0)	58 (14.8)	89,689 (7.3)
80+	10 (5.0)	15 (7.7)	25 (6.4)	64,551 (5.3)
**Race - no. (%)**				
Asian	10 (5.0)	3 (1.5)	13 (3.3)	45,389 (3.7)
Black or African American	38 (19.1)	58 (29.9)	96 (24.4)	157,060 (12.9)
Two or More Races	4 (2.0)	0 (0.0)	4 (1.0)	35,305 (2.9)
White	123 (61.8)	123 (63.4)	246 (62.6)	976,172 (79.9)
Unknown	24 (12.1)	10 (5.2)	34 (8.7)	0 (0.0)

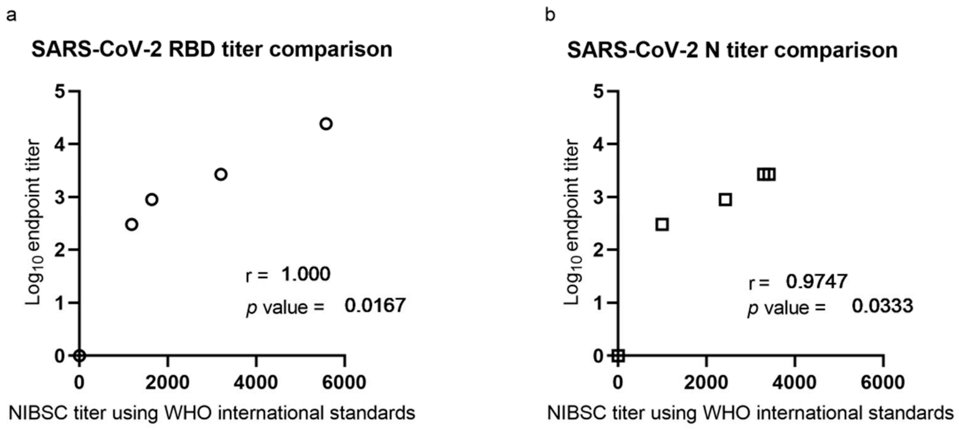

Supplemental Figure 1. Comparison of RBD and N titers between the in-house assays and that reported by NIBSC using WHO international standards. Five dots shown on each graph represent samples of one healthy donor collected before 2019 and four COVID-19 recovered patients. Spearman's Rank Correlation Coefficient r and Probability (*p*) Value (two-tailed) are shown.

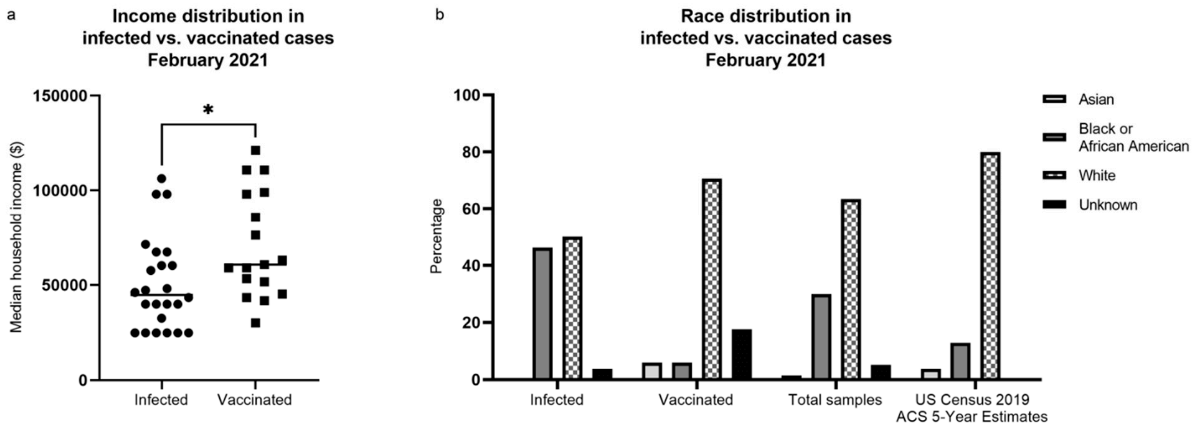

Supplemental Figure 2. Comparison of income and race between infected and vaccinated cases in February 2021. Median income distribution in infected and vaccinated groups (a): Y axis is the median household income of the area based on the zip code of the home address reported by each study subject. The line in each group represents the median. * represents a *p* value < 0.05 by an unpaired t test (two-tailed). Race distribution in infected and vaccinated groups (b): Total samples refer to all samples collected in February 2021. Data was compared to that of American Community Survey (ACS) 5-year estimates for Allegheny County, PA, generated by United States Census Bureau.
